# Intra-Individual Variability of Physical Activity in Older Adults With and Without Mild Alzheimer’s Disease

**DOI:** 10.1371/journal.pone.0153898

**Published:** 2016-04-20

**Authors:** Amber Watts, Ryan W. Walters, Lesa Hoffman, Jonathan Templin

**Affiliations:** 1 Department of Clinical Psychology, KU Alzheimer’s Disease Center, University of Kansas, Lawrence, Kansas, United States of America; 2 Department of Medicine, Creighton University, Omaha, Nebraska, United States of America; 3 Research Design and Analysis Unit, Schiefelbusch Institute for Lifespan Studies, University of Kansas, Lawrence, Kansas, United States of America; 4 Department of Educational Psychology, University of Kansas, Lawrence, Kansas, United States of America; Vanderbilt University, UNITED STATES

## Abstract

Physical activity shows promise for protection against cognitive decline in older adults with and without Alzheimer’s disease (AD). To better understand barriers to adoption of physical activity in this population, a clear understanding of daily and weekly activity patterns is needed. Most accelerometry studies report average physical activity over an entire wear period without considering the potential importance of the variability of physical activity. This study evaluated individual differences in the amount and intra-individual variability of physical activity and determined whether these differences could be predicted by AD status, day of wear, age, gender, education, and cardiorespiratory capacity. Physical activity was measured via accelerometry (Actigraph GT3X+) over one week in 86 older adults with and without AD (*n* = 33 and *n* = 53, respectively). Mixed-effects location-scale models were estimated to evaluate and predict individual differences in the amount and intra-individual variability of physical activity. Results indicated that compared to controls, participants with AD averaged 21% less activity, but averaged non-significantly greater intra-individual variability. Women and men averaged similar amounts of physical activity, but women were significantly less variable. The amount of physical activity differed significantly across days of wear. Increased cardiorespiratory capacity was associated with greater average amounts of physical activity. Investigation of individual differences in the amount and intra-individual variability of physical activity provided insight into differences by AD status, days of monitor wear, gender, and cardiovascular capacity. All individuals regardless of AD status were equally consistent in their physical activity, which may have been due to a highly sedentary sample and/or the early disease stage of those participants with AD. These results highlight the value of considering individual differences in both the amount and intra-individual variability of physical activity.

## Introduction

Physical activity shows promise for protection against cognitive decline in older adults with and without dementia [1–3]. Older adults spend about 60% of waking time in sedentary activities [4–5]. They rarely engage in structured exercise or high-intensity physical activity [6], and individuals with Alzheimer’s disease (AD) are even less active [7]. Yet few studies have directly compared physical activity among older adults with and without AD. A 2012 review of 134 studies of activity monitoring in older adults found only 4 studies of individuals with AD [8].

Although many studies have measured the average amount of physical activity, few have described patterns of variation of activity in older adults [9–11]. It would be beneficial to understand hourly, daily, and weekly activity patterns upon which interventions might be targeted. For example, if most long bouts of sitting occur in the evening on weekdays, interventions might target this time of day, perhaps modifying time spent watching television.

Most studies of objective physical activity monitoring only report the average estimated energy expenditure, average minutes per day of activity, or total time in physical activity minutes per day over an entire wear period [8] even though most activity monitors produce data as frequently as a one-second interval. Reporting average physical activity overlooks the importance of patterns of *variability* in activity levels within and across days. In a comprehensive review, fewer than 10% of studies reported on activity patterns within a day, across different days of the week, or in different seasons [8]. For instance, Cavanaugh et al. [12] reported that relative to younger adults, healthy older adults did not differ in the number of daily steps or total daily minutes of activity. However, minute-to-minute variation in ambulatory activity was lower in healthy older adults, and even lower in older adults with functional limitations, suggesting a narrower range of ambulatory behavior. Thus, patterns of variation in older adults with and without health limitations may be a useful supplemental indicator of physical activity.

**Fig 1:**
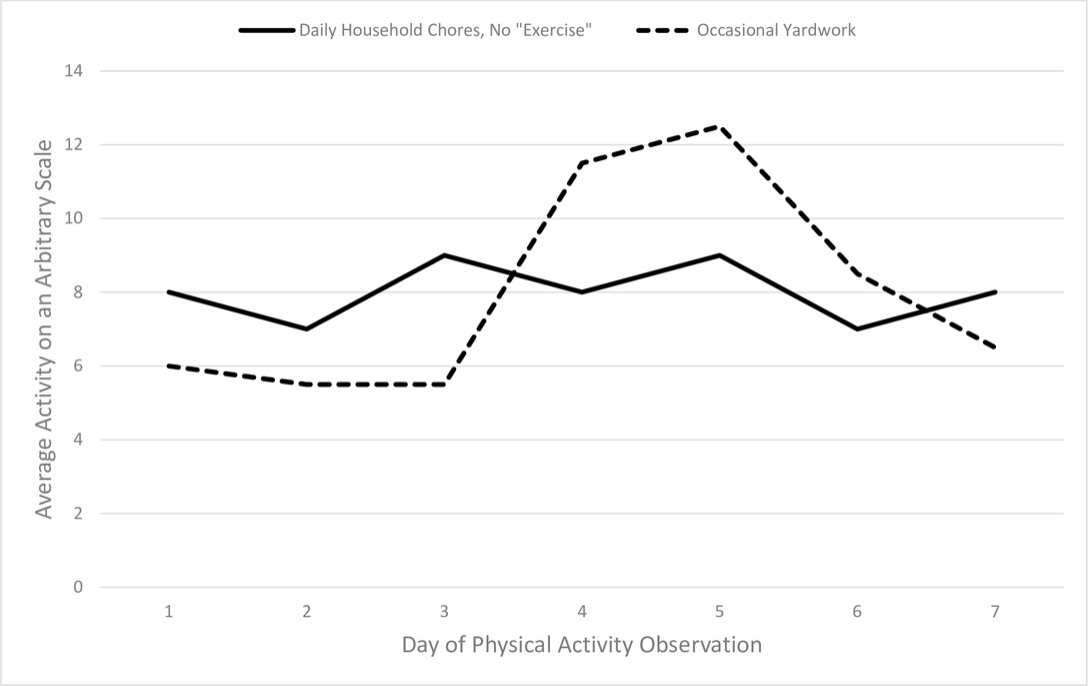
Example Activity Patterns with Same Means and Different Variability Patterns. The solid line represents example pattern 1) a retired person who does housework on most days, but who never engages in structured exercise. The dashed line represents example pattern 2) an older person who spends most of the day using the computer, but has bouts of vigorous outdoor gardening and home repairs when the weather allows.

For example, consider the following descriptions of two hypothetical older adults: one who does housework on most days, but who never engages in structured exercise, and another who spends most of the day using the computer, but has bouts of vigorous outdoor gardening and home repairs when the weather allows. These patterns are depicted in Fig 1. The type of pattern and degree of variation observed may be related to individual characteristics (e.g., sex, occupation, health status) and may also be associated with different health risks and benefits. It remains unclear whether adding a single bout of moderate to vigorous intensity activity to a day mostly filled with sitting has greater benefits than engagement in light intensity activity distributed throughout the entire day. A growing body of research describes the harms of prolonged sitting time, even in individuals who also engage in structured exercise activities [13–16]. Information about variability may be especially important in older adult populations who are highly sedentary and who rarely engage in moderate to vigorous physical activity [6].

The first and second example activity patterns (indicated by the solid and dotted lines in Fig 1, respectively) highlight what remains unknown about the comparative benefits of consistent light activity versus infrequent vigorous activity. These examples are especially relevant to older adults and retirees. Older adults are more likely to engage in light intensity everyday activities, such as walking and housework, rather than structured formal exercise activities [17]. Our previous study of older adults with and without AD found that a majority of older adults participated only in unstructured and low intensity physical activities, and those with AD did so at an even lower rate [7]. Walking and household chores were related positively to cardiorespiratory capacity, speed of physical performance, and body composition, suggesting that unstructured but utilitarian physical activity may be beneficial. Although moderate to vigorous physical activity may offer more health benefits than light intensity activity, several studies provide evidence that accumulated light intensity physical activity is associated with health outcomes in community-dwelling older adults [18–19]. Further, Buman et al. [19] reported that replacing 30 minutes per day of sedentary time with equal amounts of light physical activity was associated with higher scores on several indicators of physical health and psychosocial wellbeing.

Traditionally, studies of physical activity monitoring have summed or averaged an individual’s activity across the full period of wear, and then compared the average amount of activity between individuals with differing characteristics or used intensity cut points to compare them to weekly physical activity guidelines [20–21]. However, as illustrated in Fig 1, it is possible that two individuals with identical amounts of activity over a given week could have achieved those average amounts by drastically different patterns of activity [22]. For instance, in individuals with AD, agitation and wandering might be measured as increased movement at certain times of day. But these problematic behaviors might result in the same total amount of activity as a more structured physical activity routine, such as regular walking, which has been shown to reduce agitation as well as improve mood and sleep [23]. Thus, focusing solely on total or average amounts of activity overlooks important information about the manner in which the physical activity is accumulated. Insight into the pattern of activity can be obtained by simultaneously evaluating the amount and intra-individual variability (IIV; or, inconsistency) of an individual’s activity. Therefore, to better understand the role of within-person variation in activity levels, the present study evaluated both amount of activity and patterns of variability of physical activity in older adults with and without early stage AD over one week.

Our study had two aims. First, we determined whether some individuals moved more than others on average, and whether such individual differences could be uniquely predicted by AD status after controlling for day of monitor wear, age, sex, years of formal education, body mass, and cardiorespiratory capacity. Although one might assume that individuals with AD are less active in general than non-impaired controls, this has rarely been tested empirically.

The second aim was to quantify and predict individual differences in IIV (inconsistency) of physical activity across days. That is, we examined to what extent physical activity was more variable as a function of AD status after controlling for the aforementioned predictors. Limited information is available with which to guide hypotheses about variability of activity in individuals with AD. Studies have shown increased variability in physical and cognitive performance with advancing age and in individuals with dementia [24–26]. By contrast, Cavanaugh et al. [12] found that older adults had less minute-to-minute variation in ambulatory activity than younger adults. Thus, our study provides new insight into the role of variation in activity for older adults with and without mild AD.

## Methods

### Participants

Participants were 86 community dwelling older adults, ages 60 to 92 years, with or without mild Alzheimer’s disease *(n* = 33 mild AD, *n* = 53 non-impaired). Participants were recruited from the University of Kansas Alzheimer’s Disease Center Registry (KU-ADC), a large registry of well-characterized AD patients and older adult controls without cognitive impairment who had undergone a full physical exam, neurological testing, and a review of medical history before being recruited into the present study. Participants with mild AD had clinical dementia rating (CDR) [27] scale scores of 0.5 (very mild) or 1 (mild); non-impaired participants had CDR scores of 0 (no dementia). Participants with mild AD were required to have a study partner who would be with the participant daily (at least 10 hours per week) during the week-long activity monitoring. We excluded individuals who were confined to a bed or a wheelchair, who had inadequate visual or auditory capacity to complete study procedures, with active (< 2 years) ischemic heart disease, and those with insulin-dependent diabetes mellitus. Individuals with well-controlled, non-insulin dependent diabetes were included.

### Study Procedures

A comprehensive clinical research evaluation and review of the medical record was conducted by experienced KU-ADC study clinicians who are trained in dementia assessment and clinical research. Diagnostic criteria for AD require the gradual onset and progression of impairment in memory and in at least one other cognitive and functional domain (NINCDS-ADRDA criteria [28]. Written informed consent was provided by the participant and/or the participant’s legally acceptable representative. All study procedures were approved by the Human Subjects Institutional Review Board at the University of Kansas Medical Center.

At the baseline visit, all participants underwent assessment of vital signs, completed questionnaires, and were educated on the use of triaxial accelerometers (Actigraph GT3X+, Pensacola, FL) and daily activity monitoring logs. Control participants and the study partners of participants with AD were asked to complete daily logs to monitor their activities, the removal of the accelerometer and reasons for removal, and bedtime and waking time. The accelerometers were compact, very light weight, unobtrusive, and worn on the dominant hip secured by an elastic waist belt. Participants were instructed to wear the units 24 hours a day for seven days or until they returned to the clinic for a follow-up visit. We chose a seven-day wear period based on previous research suggesting this duration results in the reliable estimation of habitual physical activity and to allow comparison to the majority of other studies of accelerometry in the literature [29, 30]. At the follow-up visit, participants or the study partners returned the monitors, reviewed the activity logs with study staff, and completed questionnaires; participants also underwent VO_2_ max testing and received compensation.

### Measures

#### Physical activity

To assess physical activity, we used activity counts from the Actigraph GT3X+ accelerometer that measure physical movement in the medio-lateral (ML; front-to-back), antero-posterior (AP; side-to-side), and vertical (VT; rotational) axes quantified into a single tri-axial composite metric known as *average vector magnitude*, calculated as $VM=\sqrt{{ML}^{2}+{AP}^{2}+{VT}^{2}}$ [31]. Vector magnitude was chosen, as opposed to any uni-axial metric, to evaluate whether this tri-axial metric provided insight into the *total movement* of traditionally sedentary individuals (e.g., movements while seated). Average vector magnitude was aggregated every 60 minutes during waking hours as determined by the participant’s or study partner’s reported diary data. We chose to use 60-minute intervals because our sample was highly sedentary and we believed that this interval would accurately reflect total amount and variability of movement without significant loss of information.

#### Additional predictors

Age, sex, and years of formal education were reported by either the participant or study partner. Whole body mass was determined using a digital scale accurate to ±0.1kg (Seca Platform Scale, Seca Corp., Columbia, MD), and height (in cm) was measured by stadiometer with shoes off, from which body mass index (BMI; weight (kg) / height (m^2^)) was then calculated. Cardiorespiratory capacity (VO_2_ max) was measured by a graded treadmill exercise test using a modified Bruce protocol [32] designed for older adults, in which participants began walking at a pace of 1.7 miles per hour at 0% incline, and the grade and/or speed was increased at each subsequent 2-minute interval. Participants were attached to a 12-lead electrocardiograph (ECG) to continuously monitor heart rate and rhythm. Expired gases were collected continuously and oxygen uptake and carbon dioxide production were averaged at 15-second intervals (TrueOne 2400, Parvomedics, Sandy, UT). Finally, as we were interested in the variation of activity across days of monitor wear, we included contrasts for differences by day of wear (i.e., day 1 to 7), which was more relevant than day of the week, given that participants were allowed to enter the study on any day of the week.

### The Mixed-Effects Location-Scale Model

To address our specific aims of quantifying and predicting individual differences in both the amount and IIV of physical activity, we estimated the *mixed-effects location-scale model* [33, 34]. Although this model has been used to answer mean- and variability-related research questions specific to physical activity in children [35], its novelty in being applied to physical activity in older adults—and its atypical level of complexity—warrants a brief introduction to the model.

**Fig 2:**
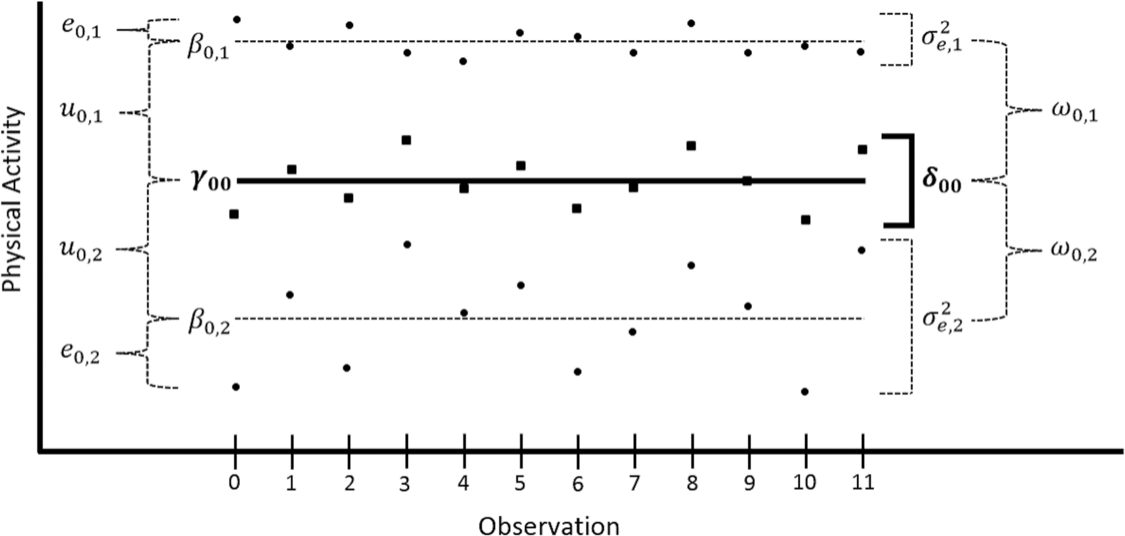
A Visual Depiction of the Mixed-Effects Location-Scale Model. Effects on the left side are for the location model; effects on the right side are for the scale model for the residual variance. Curly brackets represent differences; square brackets represent (approximate) residual variance. Dashed lines represent participant-specific effects; solid lines represent sample average (or fixed) effects. Dots represents participant-specific observed physical activity; squares represent the average of the dots. *γ*_00_ = location-model fixed intercept. *β*_0,*i*_ = participant-specific location-model intercept. *e*_0,*i*_ = participant-specific residual value. *u*_0,*i*_ = participant-specific location-model random intercept value (*β*_0,*i*_*−γ*_00_). *δ*_00_ = fixed intercept of the residual variance. *σ*^2^_*e*,*i*_ = participant-specific residual variance. *ω*_0,*i*_ = participant-specific scale-model random intercept value (*σ*^2^_*e*,*i*_*−δ*_00_).

The traditional multi-level (mixed-effects) model is used to account for the correlation (dependency) among repeated observations from the same participant. It typically assumes constant (homogeneous) IIV through the estimation of a single residual variance across all participants—here, this would assume that physical activity over the seven days was *equally inconsistent* across all participants. We tested this assumption by estimating a mixed-effects location-scale model to quantify and predict individual differences in both the amount and the IIV of physical activity by participant variables such as AD status. Modeling IIV in this manner allows variability-related research questions to be answered that would otherwise have been ignored by the traditional multi-level model. As the name implies, the mixed-effects location-scale model allows both fixed and random effects in the *location model* (aka, mean- or amount-side) and/or the *scale model for the residual variance* (aka, IIV or residual variance-side). A visual depiction of the mixed-effects location-scale model is presented in Fig 2 for two hypothetical participants (note that for the full sample there would be as many dashed lines as participants included in analysis).

As in traditional mixed-effects models, the location side of the mixed-effects location-scale model quantifies and predicts individual differences in the *mean amount* of physical activity. The unconditional (i.e., no predictor) location model is shown in (1) that corresponds to the location-model fixed and random effects presented in Fig 2.

$${PA}_{t,i}=(\gamma_{00}+u_{0,i})+e_{t,i}$$ (1)

In (1), *PA*_*t*,*i*_ represents the observed physical activity measurement at occasion *t* for participant *i*. The fixed intercept *γ*_00_ represents the average physical activity taken across all participants. The location-model random effect *u*_0,*i*_ is the deviation from the fixed intercept for participant *i* (whose variance across participants quantifies individual differences in the mean amount of activity). Finally, the residual *e*_*t*,*i*_ represents the deviation between the observed and model-predicted physical activity (here, the person-specific intercept, *β*_0,*i*_) at occasion *t* for participant *i*. The observed individual differences in the amount of physical activity, quantified by location-model random intercept variance, can then be explained by including fixed effects for individual-level predictors in the location model, such as by participant AD status (i.e., 0 = no AD, 1 = mild AD), as shown in (2).

$${PA}_{t,i}=(\gamma_{00}+u_{0,i})+\gamma_{01}(AD_{i})+e_{t,i}$$ (2)

In (2), the fixed intercept *γ*_00_ is now conditional representing the average amount of physical activity specifically for participants with no AD, whereas the fixed effect for AD status *γ*_01_ represents the mean difference in physical activity for participants with mild AD; all other effects are interpreted as in (1).

Analogously, the log-linear scale model for the residual variance quantifies and predicts individual differences in IIV (i.e., the inconsistency of physical activity) as shown for an unconditional model in (3) that corresponds directly onto the fixed and random effects for the scale model for the residual variance shown in Fig 2.

$$log(\sigma_{e_{i}}^{2})=\delta_{00}+\omega_{0,i}$$ (3)

In (3), *σ*^2^_*e*,*i*_ is the residual variance (or IIV) for participant *i*, the natural log of which is predicted directly instead to ensure positive predicted residual variances (Harvey, 1976). Given this log-linear model is unit-specific [36], the fixed intercept *δ*_00_ represents the log residual variance specifically for an individual with *ω*_0,*I*_ = 0, in which the scale-model random effect *ω*_0,*i*_ represents the deviation from the fixed intercept for the log residual variance for participant *i* (whose variance across participants quantifies individual differences in the inconsistency of activity). Conceptually similar to the location model, the observed individual differences in the IIV of physical activity, quantified by scale-model random intercept variance, can then be explained by including fixed effects of individual-level predictors in the scale model for the residual variance, such as by participant AD status, as shown in (4).

$$log(\sigma_{e_{i}}^{2})=(\delta_{00}+\omega_{0,i})+\delta_{01}(AD_{i})$$ (4)

In (4), the fixed intercept is now the log of the residual variance specifically for an individual with mild AD whose scale-model random intercept *ω*_0,*I*_ = 0, whereas the fixed effect for AD status *δ*_01_ represents the difference in the log residual variance for participants with mild AD, in which a positive *δ*_01_ would indicate more variable (i.e., more inconsistent) physical activity for participants with AD.

### Statistical Analysis

Estimating the mixed-effects location-scale model can be problematic using maximum likelihood (ML) such as when using SAS’s PROC NLMIXED [35]. Instead, all models were estimated using a custom-built Markov chain Monte Carlo (MCMC) estimator in R software; details of this estimator are available from author RWW. Data from all participants collected during the first seven full days of monitor wear were considered for analysis; no imputation methods were needed for missing occasions. Given its skewed residuals, the average vector magnitude outcome was natural log transformed. A set of preliminary single-predictor models were estimated initially to determine which predictors to include in the final mixed-effects location-scale model; any predictor for which the 80% credible interval excluded zero (akin to *p* < .20) was included. The significance of individual differences (i.e., random effect variances) in the location and scale models was evaluated using the deviance information criterion (DIC; in which smaller is better). The significance of fixed effects was determined by 95% credible intervals that excluded zero (akin to *p* < .05).

## Results

Average vector magnitude was measured between 5 and 13 days (*M* = 124.76, *SD* = 129.10, range = 0.00 to 1734.20). Of the 100 participants enrolled, 92 had valid Actigraph data and provided 9,238 total observations. Of these observations, 291 (3.12%) with a recorded average vector magnitude in a given epoch of 0 were excluded (i.e., a presumably awake participant did not move in any axis during the 60-minute epoch, which was considered unlikely) and an additional 84 (0.94%) observations were excluded because they occurred after the seventh day of wear. In addition, within the AD group, five participants were missing their baseline cardiorespiratory capacity (i.e., VO_2_ max) measurements and one participant was missing years of education. No predictors were missing within the control group. Therefore, the final mixed-effects location-scale model was based on 8,429 total observations from 53 healthy controls and 33 participants with mild AD. Thirty-three participants (38.37%) had seven days of accelerometer wear, 43 (50.00%) had six days of wear, and 10 (11.63%) had five days of wear. These participants averaged approximately 98 occasions across all days of wear (*SD* = 12.50, range = 58 to 120), and averaged between 14 and 16 occasions across days (range of occasions for a given day = 8 to 20). Although healthy controls averaged significantly more occasions than participants with mild AD (healthy control *M* = 100.98 vs. mild AD *M* = 93.36), *t*(84) = 2.86, *p* < .05, there was no significant between-group difference in the number of days of wear, *Χ*^2^ (2, *N* = 86) = 4.79, *p* = .091. In addition, the control group had significantly more women (*n* = 37, 69.81%) than the AD group (*n* = 10, 30.30%), *Χ*^2^ (1, *N* = 86) = 12.81, *p* < .001. Table 1 provides additional descriptive statistics by group, in which the control group was more educated (although both groups averaged an equivalent of a bachelor’s degree), *t*(84) = 2.40, *p* < .05; the groups were similar in age, VO_2_ max, and BMI.

**Table 1:** Group-Specific Descriptive Statistics for Participants included in the Final Model.

	Healthy Individuals (*n* = 53)		Individuals with Mild AD (*n* = 33)	
	*M* (*SD*)	Range	*M* (*SD*)	Range
Age	73.19 (6.53)	62 to 92	72.73 (7.47)	60 to 86
Years of Education	17.32 (3.38)	12 to 25	15.61 (2.94)	10 to 20
VO_2_ max	1.60 (0.45)	0.79 to 2.74	1.73 (0.56)	0.62 to 3.10
Body Mass Index	26.42 (4.42)	19.69 to 36.68	27.18 (5.03)	19.31 to 38.38

The average vector magnitude across all observations was 131.89 (*SD* = 130.72, range = 0.10 to 1734.20), indicating that the final sample was generally involved in sedentary activities. Significant individual differences in (the log of) average vector magnitude were indicated by the improvement of fit of an unconditional location-model with a random intercept (i.e., a traditional multi-level, mixed-effects model without any predictors) over a single-level model (DIC = 28,051.44 and 29,723.48, respectively). The location-model random intercept variance predicted 95% of participants to have average vector magnitude between 43.54 and 145.78. This indicated that even after removing observations in which participants did not move at all, nearly every participant in the sample spent most of their time engaged in sedentary behaviors.

The final location model is shown in (5), in which BMI was excluded, age was centered at 70, education was centered at 16 years (i.e., a college graduate), and cardiorespiratory capacity (VO_2_ max) was centered at 1.50 mL/(kg·min) to create an interpretable intercept.

$$log({VM}_{t,i})=(\gamma_{00}+u_{0,i})+\gamma_{20}(Day2_{t,i})+\gamma_{30}(Day3_{t,i})+\gamma_{40}(Day4_{t,i})+\gamma_{50}(Day5_{t,i})+\gamma_{60}(Day6_{t,i})+\gamma_{70}(Day7_{t,i})+\gamma_{01}({AD}_{i})+\gamma_{02}({Age}_{i}-70)+\gamma_{03}({Ed}_{i}-16)+\gamma_{04}({Woman}_{i})+\gamma_{05}({VO2}_{i}-1.50)+e_{t,i}$$ (5)

When compared to the final location model in (5), including individual differences in IIV of average vector magnitude improved model fit (DIC = 25,399.39 vs. 26,392.28, respectively), indicating that some participants were more consistently sedentary in their physical activity, whereas others were more variable. The preliminary scale models for the residual variance indicated that neither BMI nor VO_2_ max predicted the inconsistency of physical activity; thus, the final scale model for the residual variance is shown below in (6).

$$log(\sigma_{e_{t.i}}^{2})=\left(\delta_{00}+\omega_{0,i}\right)+\delta_{20}(Day2_{t,i})+\delta_{30}(Day3_{t,i})+\delta_{40}(Day4_{t,i})+\delta_{50}(Day5_{t,i})+\delta_{60}(Day6_{t,i})+\delta_{70}(Day7_{t,i})+\delta_{01}({AD}_{i})+\delta_{02}({Age}_{i}-70)+\delta_{03}({Ed}_{i}-16)+\delta_{04}({Woman}_{i})$$ (6)

Table 2 presents the results of the final mixed-effects location-scale model that simultaneously estimated both the location model in (5) and the scale model for the residual variance in (6). With respect to the location model, compared to control participants, those with AD averaged significantly less average physical activity by 20.55%, ([1 –exp(–0.23)]*100), *γ*_01_ = –0.23, 95% CI [–0.45,–0.02], pseudo-*R*^2^ = 1.89%, after controlling for day of wear, age, education, sex, and cardiorespiratory capacity. In addition, greater cardiorespiratory capacity predicted a greater amount of movement, such that a 1/10^th^ increase in VO_2_ max was associated with 2.63% greater average movement, (0.26/10 = 0.026; [exp(0.026)– 1]*100). Thus, participants with higher fitness levels were likely to be more active. Further, the average amount of movement differed by day of wear, such that there was 17.30% less movement on day 7 than day 1, ([1 –exp(–0.19)]*100). This result suggests that averaging activity across days may overlook nuances identified by evaluating each day of wear separately.

**Table 2:** Results of the Final Mixed-Effects Location-Scale Model.

			95% Credible Interval	
Location Model	Posterior Mean	Posterior SD	Lower	Upper
Fixed Intercept	4.37	0.14	4.11	4.62
Day of Wear^a^				
Day 2	–0.03	0.04	–0.11	0.04
Day 3	–0.05	0.04	–0.12	0.02
Day 4	–0.06	0.04	–0.13	0.01
Day 5	0.01	0.04	–0.07	0.08
Day 6	–0.03	0.04	–0.10	0.04
Day 7	**–0.19**	0.05	–0.29	–0.09
Age (0 = 70)	–0.01	0.01	–0.03	0.01
Years of Education (0 = 16)	0.01	0.02	–0.02	0.05
Woman	0.18	0.14	–0.09	0.44
VO_2_max (0 = 1.50)	**0.26**	0.10	0.07	0.46
Mild Alzheimer's Status	**–0.23**	0.11	–0.45	–0.02
Scale Model for the Residual Variance				
Residual Variance Fixed Intercept	0.19	0.12	–0.02	0.44
Day of Wear^a^				
Day 2	**0.16**	0.05	0.06	0.26
Day 3	**0.14**	0.05	0.04	0.23
Day 4	0.08	0.05	–0.02	0.19
Day 5	**0.12**	0.05	0.01	0.23
Day 6	–0.01	0.05	–0.10	0.09
Day 7	**0.39**	0.07	0.25	0.54
Age (0 = 70)	0.01	0.01	–0.01	0.02
Years of Education (0 = 16)	–0.02	0.02	–0.06	0.01
Woman	**–0.28**	0.12	–0.54	–0.10
Mild Alzheimer's Status	0.01	0.11	–0.21	0.22
Variance Components				
Location-Model Random Intercept	**0.26**	0.05	0.18	0.37
Location-Scale Random Intercept Correlation	**–0.15**	0.04	–0.23	–0.09
Scale-Model Random Intercept	**0.26**	0.05	0.19	0.38

^a^Reference day was study day 1.

*Note*. Statistically significant effects are presented in boldface. DIC = 25,396.46. Age was centered at 70, years of education was centered at 16 (i.e., a college graduate), and cardiorespiratory capacity (VO_2_max) was centered at 1.50 mL/(kg·min).

**Fig 3:**
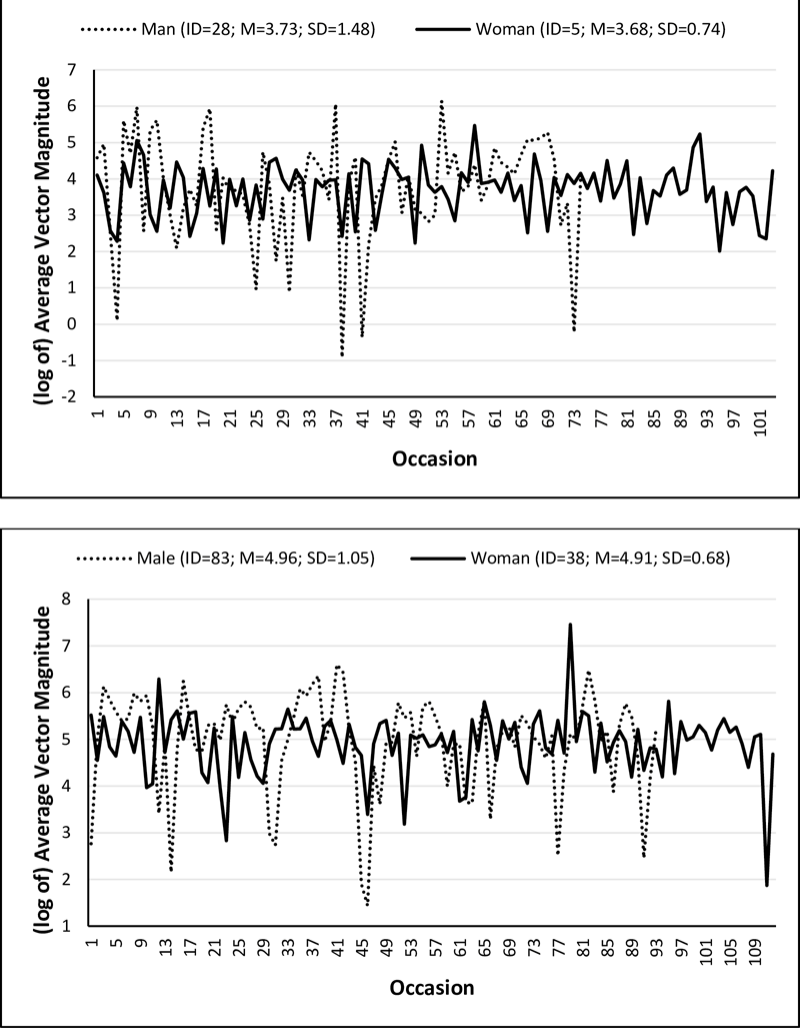
Between-Sex and Between-AD Status Differences in the Variability of Physical Activity Across Occasions. Man and woman with mild AD (top) and a healthy man and woman (bottom).

With respect to the scale model for the residual variance, the movement of participants with AD was only 0.54% more variable than control participants, ([exp(0.01)– 1]*100), *B* = 0.01, 95% CI [–0.21,0.22], after controlling for day of wear, age, education, and sex. Thus, although control participants moved more on average than participants with mild AD, individuals within each group had equally consistent levels of physical activity. Interestingly, the physical activity of women was 24.42% more consistent compared to men, ([1 –exp(–0.28)]*100). That is, although the *amount* of physical activity was similar between men and women, women maintained a *more consistent* level of physical activity. This between-sex difference in IIV is shown in Fig 3, in which (the log of) physical activity is presented for two participants with mild AD in the top plot and two healthy controls in the bottom plot. The participants within each plot average similar amounts of physical activity, but the physical activity of women in both groups is more consistent. Further, significant fluctuations in variability were observed across days of wear, again pointing to the need to evaluate patterns of activity by day of wear, rather than assuming equivalent amounts and variability in movement across days.

Finally, the significant correlation between the location-model and scale-model random intercepts of –0.15 indicated that participants who averaged more physical activity tended to be more consistent (i.e., less variable) in their physical activity. That is, moderately active participants stayed active, whereas sedentary participants were mostly sedentary with occasional bouts of activity.

## Discussion

Few studies have addressed within-person daily variation in physical activity among older adults. Our study is the first to describe differences between individuals with and without mild AD in both the amount *and* consistency of activity. In contrast to the typical practice of averaging physical activity data over multiple days of wear, our results highlight the importance of considering individual differences in both the amount and IIV of physical activity and provide insight as to what extent these individual differences could be predicted by AD status, gender, cardiorespiratory capacity, and different days of the week-long monitoring period.

Although the average amount of physical activity was 21% lower in participants with mild AD compared to those without, both groups were similarly consistent in their activity. Given that the majority of our sample was sedentary (even after removing observations with zero movement), increasing the amount of physical activity will likely also increase the amount of variability (i.e., create variation relative to a lack of activity). Thus, in primarily sedentary individuals, increased variability is likely to be associated with improved health outcomes. Although participants with AD moved less on average, only a small proportion of individual differences in average amount of physical activity was explained by AD status and the other predictors. Thus, most of the reasons for individual differences in the amount of physical activity remained unexplained. In early stage AD, physical function remains relatively preserved, such that agitation or wandering are less likely than in later stages of the disease. Studies including individuals with more advanced stages of AD might find that the individual differences in the amount of physical activity predicted to a greater extent by AD status, duration, or severity.

Evaluating individual differences in IIV provided novel insight into daily activity patterns and differences in the consistency of activity between older men and women. In the introduction we posed two example physical activity patterns in which individuals had the same mean activity level, but different patterns of variability. Both hypothetical activity patterns describe what we observed in the activity means and variabilities in our sample. That is, men and women did not differ in their average amount of physical activity, but women maintained a more consistent level of activity, whereas men had a larger magnitude of fluctuation between active and sedentary periods. This is consistent with a previous study of older adults reporting that women accumulated less sedentary time than men and broke it up more often [37]. Previous research has also found that much of women’s daily activity is made up of household chores, caring for others, running errands, and other domestic work [38, 39] which may result in different activity patterns than women or men who report fewer of these types of activities. Given the current findings of gender differences in the variability of activity, the sources of such differences may be better understood by explicitly including household chores and other unstructured physical activity to account for the full range of activity patterns of both women and men.

An important question raised by these findings is the extent to which variability in physical activity may be beneficial or harmful. We found that the amount of physical activity was negatively correlated with the variability of activity, such that more active participants tended to be more consistent in their activity. However, there is limited evidence to date regarding the association between patterns of activity variation and healthier outcomes, perhaps in part because variability in activity is frequently tied to the amount of activity, especially in sedentary individuals. For example, in a very sedentary individual, any amount of change in activity may be an improvement upon constant sitting, which would necessarily increase their IIV of physical activity. But an individual who is consistently active at a low level may have healthier outcomes than someone who fluctuates frequently between sitting and activity in short bouts. These questions have yet to be addressed in the majority of the literature on physical activity.

Not surprisingly, we found that participants with a higher cardiorespiratory capacity (VO_2_ max) averaged greater amounts of physical activity. It may be that more activity results in better cardiorespiratory fitness, or that fitter participants are more inclined to be active. Interestingly, cardiorespiratory capacity was not associated with consistency of physical activity, suggesting that that multiple patterns of activity could result in greater fitness levels (i.e., both highly consistent and highly variable weekly activity patterns may relate to higher levels of fitness). If only highly consistent patterns of activity resulted in cardiorespiratory fitness, higher VO_2_ max would have predicted lesser variability. The present study cannot extrapolate further about this relationship, however, due to the relative inactivity and low fitness levels of the participants.

The present findings also imply that future research should examine differences in the variability of physical activity across days of observation. For example, a three-level mixed-effects location-scale model described by Li and Hedeker [40], in which observations are nested within days which are nested within individuals, could quantify random between-day differences (assuming an adequate number of days) that could then be predicted by day-level characteristics, such as weekend versus weekday. Even after excluding days on which participants first received their monitors or returned their monitors (i.e., incomplete days of wear), we observed significant differences in the IIV of physical activity after the first complete day of wear, with the last full day of wear being the most variable. This result was not likely due to weekend versus weekday wear, as the day of the week on which the first study day occurred varied across participants. Thus, although we cannot explicitly state that a weekend day did not predict the amount or variability of activity, study day 1 was on the weekend for 7% of individuals with 32% of all observations occurring on a weekend day. Further, because we included any participant with a minimum of five valid days of monitor wear, it is also possible that individuals who did not complete a full seven days of monitor wear were somehow different in their activity patterns than those who completed all seven days of monitoring. It may be that wearing the monitor serves as an implicit signal to participants to temporarily modify their usual behavior to appear more active and thus socially desirable (i.e., a Hawthorne effect). As the novelty or awareness of the monitoring fades and participants grow accustomed to it, they may return to their usual activity patterns. We could not identify any studies that explicitly measured such an effect for physical activity monitoring, but this seems a plausible explanation that could be replicated in future work.

### Limitations and Future Directions

As clinical samples of individuals diagnosed with Alzheimer’s disease are difficult to obtain, our sample size was limited. As such, future studies are needed to replicate our results in order to begin building valid evidence of these findings and better inform their generalizability to the older adult population with and without mild AD. Further, additional details about acute and chronic medical conditions should be collected to help account for unexplained variance in the variability of day-to-day activity in older adults.

An important direction for future research is to assess to what extent greater degrees of variability in physical activity are associated with differential subsequent long-term health outcomes. Several studies suggest benefits of accumulation of light intensity activity [18, 19]. A recent dose-response study reported that lower doses of exercise conferred cognitive benefits, but that greater benefits were seen at higher doses of exercise only when adherence to exercise was good [41]. However, there is currently no general consensus as to whether accumulation of light intensity activity is equally beneficial in the long run to less frequent, but more moderate to vigorous activity.

In conclusion, the ability to predict future health outcomes from different patterns of physical activity is likely to be very informative. To successfully motivate older adults to change their physical activity patterns, we need evidence regarding the benefits of augmenting activity levels in different ways, such as by adding a single bout of moderate to vigorous activity to a day mostly filled with sitting, or by engaging in lighter intensity activity throughout the day. Older adults are more likely to follow behavior change regimens if that change in behavior feels achievable and fits in with individuals’ typical habits and preferences. Analysis of individual differences in daily activity patterns may offer opportunities to better tailor such interventions.
